# Supplementary material for: Deletion of Hnrnpk Gene Causes Infertility in Male Mice by Disrupting Spermatogenesis
Source: Cells. 2022 Apr 9;11(8):1277. doi: 10.3390/cells11081277 (PMC9028439; doi:10.3390/cells11081277)
Supplement: Supplementary file 1 [file cells-11-01277-s001.zip › Supplementary Figure S1-S3 and Supplementary Table S1-S2.pdf]

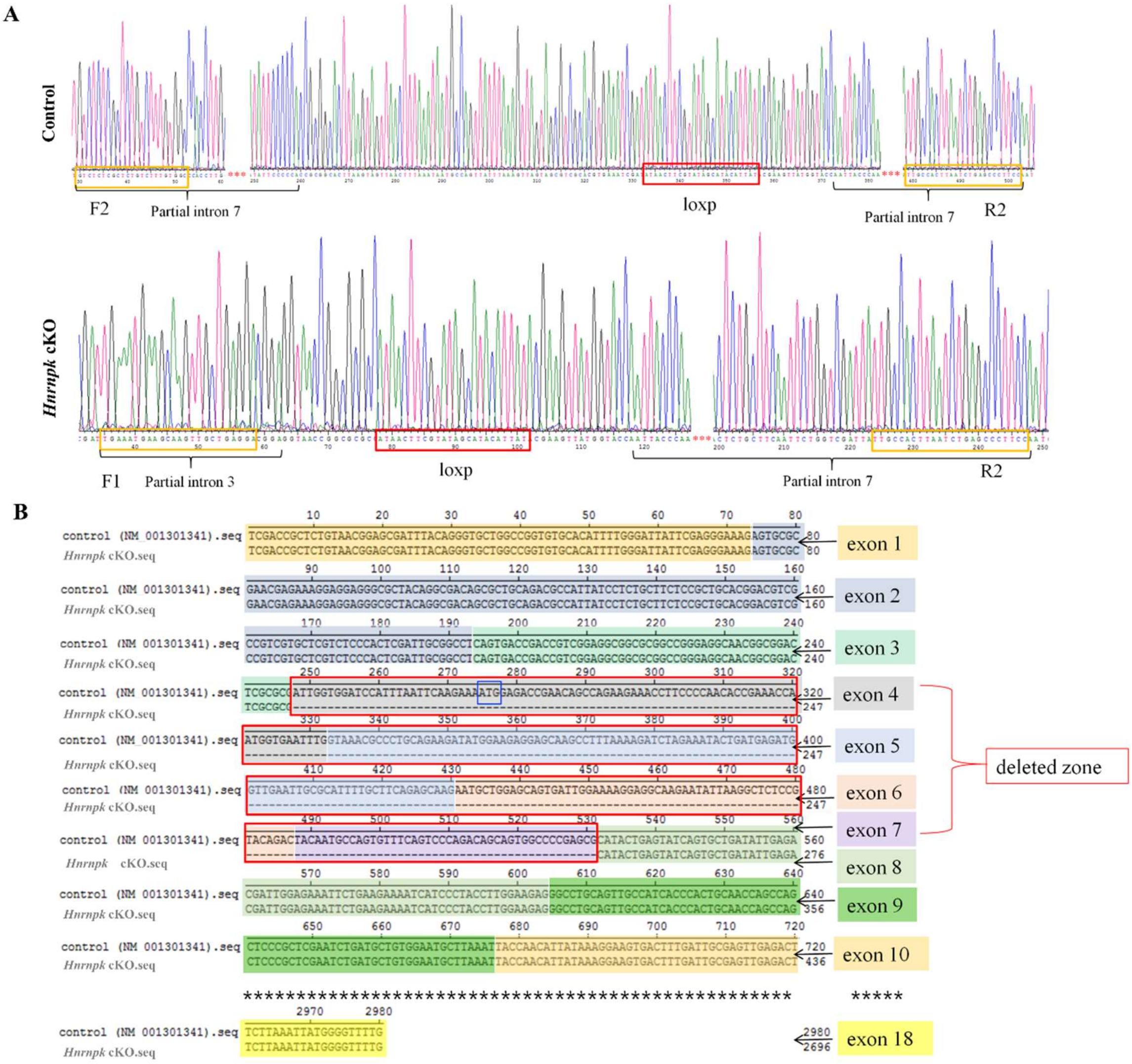

Supplementary Figure S1. DNA sequencing verified the knockout fragment of *Hnrnpk* gene. (A) Sequences of individually cloned fragments from WT control and *Hnrnpk* cKO mice genomic DNA of *Hnrnpk* gene. (B) Sequence alignment of exon 1 to exon 10 of the mouse *Hnrnpk* mRNA sequence between WT control (NM_001301341) and *Hnrnpk* cKO.

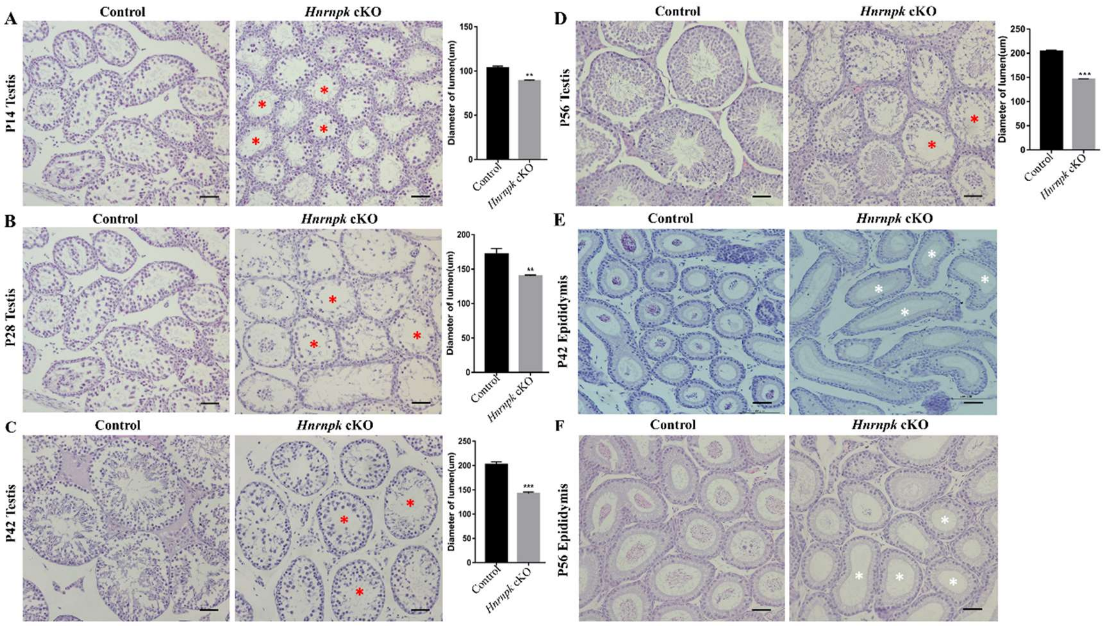

Supplementary Figure S2. HE staining of mouse testis and epididymis at different stages. (A) Typical pictures of testicular tissue morphology of P14 WT control and *Hnrnpk* cKO mice**.** (B) Typical pictures of testicular tissue morphology of P28 WT control and *Hnrnpk* cKO mice**.** (C) Typical pictures of testicular tissue morphology of P42 WT control and *Hnrnpk* cKO mice. (D)Typical pictures of testicular tissue morphology of P56 WT control and *Hnrnpk* cKO mice. (E) Typical pictures of epididymis tissue morphology of P42 WT control and *Hnrnpk* cKO mice. (F) Typical pictures of epididymis tissue morphology of P56 WT control and *Hnrnpk* cKO mice**.** The red star represents the lumen where the germ cell layer is absent, and the white asterisk indicates a noontime cluster of mature sperm in the epididymis. $P < 0.001$ (***), $P < 0.01$ (**), n = 3. Bar = 50 μm.

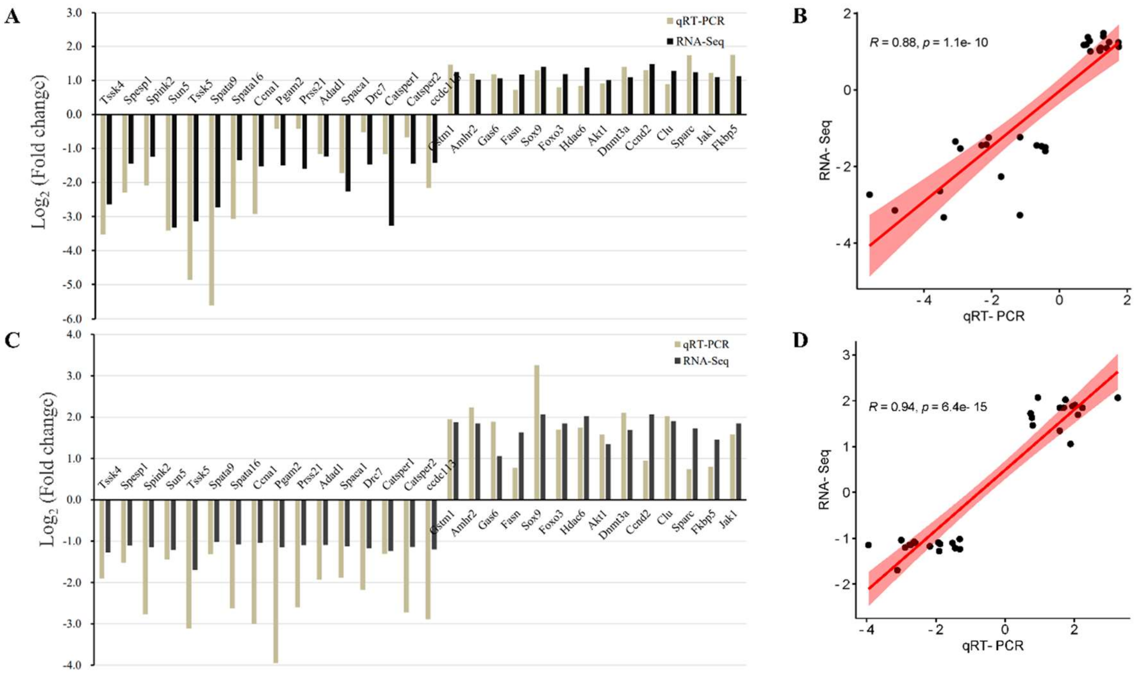

Supplementary Figure S3. Validation by qRT-PCR analysis of 30 differentially expressed genes from RNA-Seq approach. (A) The fold-change of differentially expressed genes obtained by RNA-Seq analysis and qRT-PCR analysis in P21 testes. (B) Correlation between the fold-change in RNA-Seq analysisand those obtained by qPCR analysis for the 30 selected genes in P21 testes. (C) The fold-change of differentially expressed genes obtained by RNA-Seq analysis and qRT-PCR analysis in P28 testes. (D) Correlation between the fold-change in RNA-Seq analysisand those obtained by qPCR analysis for the 30 selected genes in P28 testes.

Supplementary Table S1. Primer sequences for the specific genes of qRT-PCR analysis

Gene	Primer sequence（5'-3'）	Tm (℃)	Length （bp）	GenBank Accession No.
Tssk4	F:GTGAGATACAGGTAATGAAAGTCC<br>R: GTATACTCGGGATGTGGTCTC	60	82	XM_036158850.1
Tssk5	F: TACCTGGCCTATGCAACAC<br>R: TGATAGCCACCATAGTGTGG	60	90	NM_183099.2
Spata4	F: GCAGAGGAGTGTTTACCACTG<br>R: GGACAGAACGACTCAGGCG	60	145	NM_133711.4
Spata9	F: TCTCTGGAAGACTTGAGGG<br>R:CTGTGATAATCTTAAGATGGTAGGG	60	91	XM_030247441.2
Spata16	F: TCTACACAGATACCCAAGGAC<br>R: GTCAGCAGATATTGCTGGG	60	81	NM_001177914.1
Sun5	F: ATAGACCTCCATCCTTGGC<br>R: AACTTGCATCTCCTAGGCC	60	120	XM_030252189.2
Prss21	F:GAACTCCACGTACAAGTTTGAG<br>R: ATGGCAGACTCTCATCTTCTC	60	82	NM_020487.4
Pgam2	F: AACATCTGGAAGGGATGTCC<br>R: TCTGGTCCAGCTCATACAC	60	83	NM_018870.3
Adad1	F: GCAGTATACTTGTTGGTGATGG<br>R: GTGAGTGCGTCATCAACAC	60	84	NM_009350.3
Ccna1	F: TCAAATGCTCAGCAGAGCT<br>R: TGATTGCTGTGATCTCCTGG	60	115	XM_030252400.1
Spaca1	F: ACACTTGGATTCACCTCCA<br>R: ACACTTGGATTCACCTCCA	60	125	NM_001290443.1
Drc7	F: GATCACTCGGTCATAAGCCA<br>R: CTCCTCCTTCAATGAGTTGGT	60	80	NM_001042715.3
Ccdc113	F: ACTCTGCTCTCAAAACAGAAACA<br>R: GCCTGGATCGTAACTGTGAAA	60	132	XM_006531000.3
Catsper 1	F: CTGCCTCTTCCTCTTCTCTG<br>R: GTGGTAAAGATGTTCTGGAAGC	60	80	NM_139301.3
Catsper 2	F: TCCGGGAGATACTTGATCCT<br>R: GCTTTATGGAGAAGCGCAC	60	80	XM_006531000.3
Spink2	F: CTTTGCAGCCTCTCATGAG<br>R: CAGGGTTGAGGTTCCTAGG	60	117	XM_011249564.4
Amhr2	F: GTACTCCCTGGTCTTGCTC<br>R: TCTTGTCCAAGAGCTCTGG	60	114	NM_001356575.1
Clu	F: AGAAAGAGGATGCTCTGGAG<br>R: CCATCATGGTCTCGTTACAC	60	83	NM_013492.3

Sox9	F: CATGAACGCCTTCATGGTG<br>R: TCTTCTCGCTCTCGTTCAG	60	130	NM_011448.4
Gas6	F: CCAAATGTGTTCAGAACTTGC<br>R: TGAGGTCTTGGCAGATATGAG	60	80	NM_019521.2
Foxo3	F: CAAACGGCTCACTTTGTCC<br>R: CCGGATGGAGTTCTTCCAG	60	108	NM_019740.3
Hdac6	F: CTGGGACAATCATCACCCT<br>R: GGTAGGATGAGACAGCGAG	60	95	XM_01731838<br>9.3
Dnmt3a	F: AGACGTCTCCAACATGAGC<br>R: ATCTGATCAGGACACAAGCA	60	121	XM_00651495<br>6.4
Jak1	F: CGACATTCTCCAAAGAAGCAG<br>R: AATCATACTGTCCCTGTGCA	60	114	XM_00651495<br>6.4
Akt1	F: CTCAAGAAGGAGGTCATCGT<br>R: GTACTTGAGGGCCGTAAGG	60	104	NM_00133110<br>7.1
Sparc	F: ATTGCAAACATGGCAAGGT<br>R: TCATTGCTGCATACCTTCTC	60	117	NM_00129081<br>7.1
Gstm1	F: CTGTTCTCTGCCTCAGGAG<br>R: ACATAGGTGACCTTGTCCC	60	122	NM_010358.5
Fasn	F: CTGACTCGGCTACTGACAC<br>R: TTAGGGTAGGACCCTCAGG	60	110	NM_007988.3
Ccnd2	F: CATTGAGCACATCCTTCGC<br>R: GCAAACTTGAAGTCGGTAGC	60	106	XM_03616578<br>7.1
Fkbp5	F: GAATATGCTTATGGCTCGGC<br>R: TGAAATCAAGGAGCTCAATCTC	60	84	NM_010220.4
Hnrnpk	F: TTTGGCTGGATCTATTATTGGC<br>R: CTGCTTCACACTGTTCTGC	60	181	XM_00651710<br>4.4
18S rRNA	F: ACCGCAGCTAGGAATAATGGA<br>R: GCCTCAGTTCCGAAAACCA	60	63	NR_003278.3
Gapdh	F: AGGTCGGTGTGAACGGATTTG<br>R: GGGGTCGTTGATGGCAACA	60	95	NM_00128972<br>6.1

Supplementary Table S2. Primary antibody list

Antibody	Resource	Usage and dilution	
Anti-γH2A.X	Beyotime, AF1201	IF	1:200
Anti-hnRNPK	Abcam, ab39975	IF	1:200
	Beyotime, AF1972	WB	1:1000
Anti-PLZF	Abcam, ab104854	IF	1:200
Anti-SYCP3	Abcam, ab97672-100	IF	1:100
Anti- Ki67	BD, 550609	IF	1:200
Anti-SOX9	Beyotime, AF2329	IF	1:50
Anti-GAPDH	Beyotime, AF0006	WB	1:1000
